# Transcriptome analysis of human colorectal cancer biopsies reveals extensive expression correlations among genes related to cell proliferation, lipid metabolism, immune response and collagen catabolism

**DOI:** 10.18632/oncotarget.20345

**Published:** 2017-08-18

**Authors:** Lai Xu, Rong Wang, Joseph Ziegelbauer, Wells W. Wu, Rong-Fong Shen, Hartmut Juhl, Yaqin Zhang, Lorraine Pelosof, Amy S. Rosenberg

**Affiliations:** ^1^ Office of Biotechnology Products, CDER, FDA, Silver Spring, MD 20993, USA; ^2^ HIV/AIDS Malignancy Branch, NCI, Bethesda, MD 20892, USA; ^3^ Facility for Biotechnology Resources, CBER, FDA, Silver Spring, MD 20993, USA; ^4^ Indivumed GMBH, Hamburg 20251, Germany; ^5^ Office of Hematology and Oncology Products, CDER, FDA, Silver Spring, MD 20993, USA

**Keywords:** cell cycle, lipid metabolism, inflammation, collagen catabolism, gene co-expression network

## Abstract

Precise characterization of biological processes critical to proliferation and metastasis of colorectal cancer should facilitate the development of diagnostic and prognostic biomarkers as well as novel treatments. Using mRNA-Seq, we examined the protein coding messenger RNA (mRNA) expression profiles across different histologically defined stages of primary colon cancers and compared them to their patient matched normal tissue controls. In comparing 79 colorectal cancers to their matched normal mucosa, tumors were distinguished from normal non-malignant tissues not only in the upregulation of biological processes pertaining to cell proliferation, inflammation, and tissue remodeling, but even more strikingly, in downregulated biological processes including fatty acid beta oxidization for ATP production and epithelial cell differentiation and function. A network analysis of deregulated genes revealed newly described cancer networks and putative hub genes.

Taken together, our findings suggest that, within an inflammatory microenvironment, invasive, dedifferentiated and rapidly dividing tumor cells divert the oxidation of fatty acids and lipids from energy production into lipid components of cell membranes and organelles to support tumor proliferation. A gene co-expression network analysis provides a clear and broad picture of biological pathways in tumors that may significantly enhance or supplant current histopathologic studies.

## INTRODUCTION

Colorectal cancer is the third most common cancer and the third most common cause of cancer-related deaths in men and women in the United States [1]. An emerging theme in cancer biology is that metabolic regulation is intricately linked to cancer progression and therefore, factors that promote proliferation may also directly or indirectly promote metabolic changes to support rapid proliferation and metastasis [2]. Immunohistochemical staining [3] and microarray analyses [4] have shown that cancer cells, unlike their healthy tissue counterparts, switch from aerobic mitochondrial oxidative phosphorylation to glycolysis (The Warburg Effect) as the primary energy source even in the context of an aerobic microenvironment. Glycolysis provides cancer cells not only with energy, but also with metabolites including ribose sugars, glycerol, citrate, and nonessential amino acids which are essential for cellular proliferation [5–7].

We identified cancer specific gene expression patterns and cancer associated signaling and regulatory pathways in colorectal cancer samples as compared to their patient matched healthy colonic tissues using mRNA-Seq. Our study sought to explore expression correlations among dysregulated genes pertaining to biological pathways critical in tumor proliferation and metastasis by identifying and quantifying critical mRNA gene co-expression networks and hub genes.

## RESULTS

### Differential gene expression in colorectal cancer vs. normal intestinal mucosa

**Figure 1:**
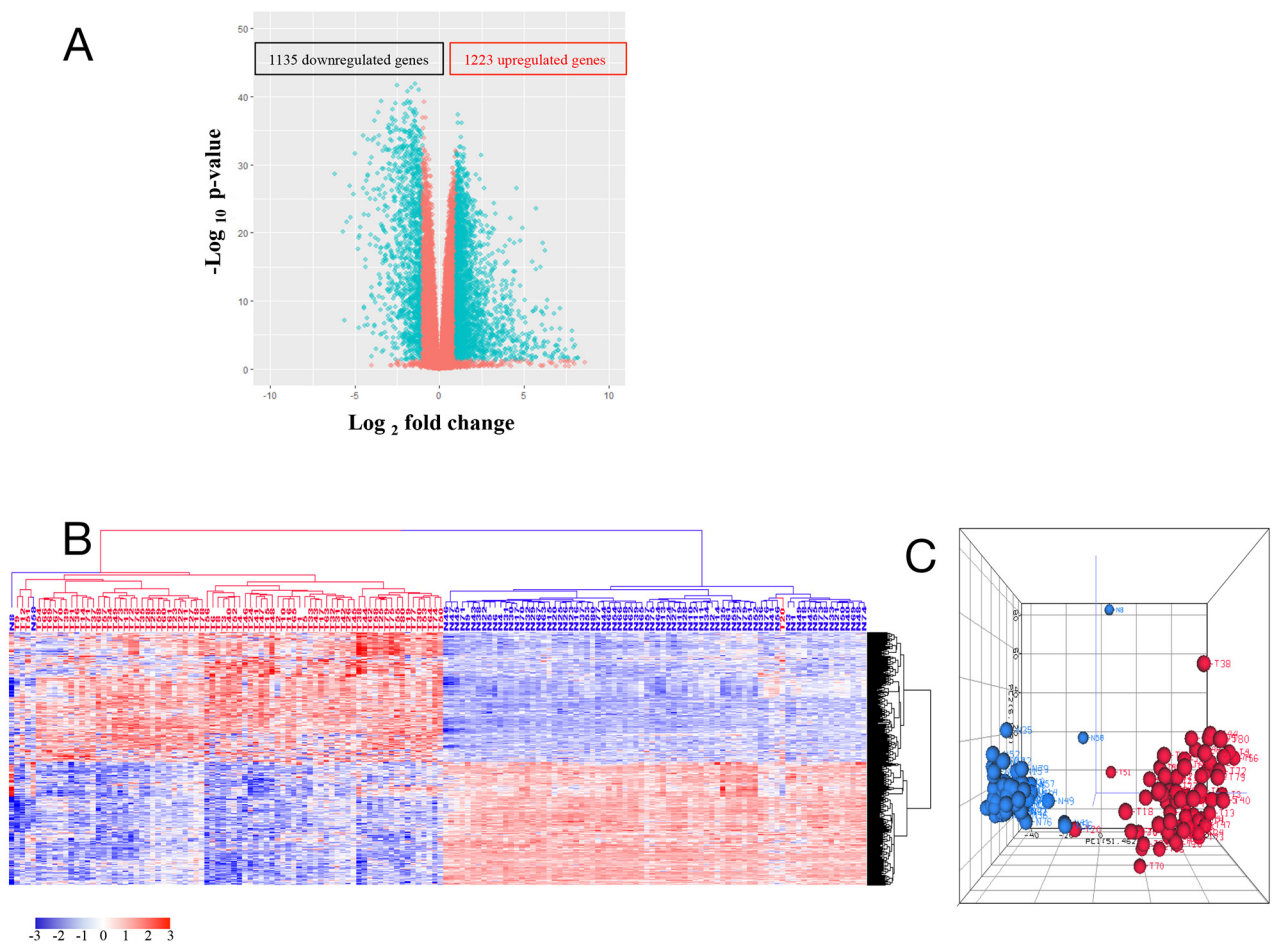
**(A)** Volcano plot of 10,255 genes with average FPKM > 1 and FDR ≤ 0.05. Each circle corresponds to one gene. The x-axis represents gene log_2_ (T/N) value, and the y-axis represents log_10_ p-value calculated from ANOVA test between tumor and normal controls. T represents gene FPKM value in tumor sample, and N represents the gene FPKM value in normal control. **(B)** and **(C)** HCA (b) and PCA (c) of 1,223 upregulated genes and 1,135 downregulated genes. Tumor is marked with red dendrogram and normal sample is marked with blue dendrogram.

To identify genes that were dysregulated in tumors compared with normal tissues, 79 tumor (T) and normal (N) paired samples were evaluated. From 10,255 genes which showed abundant expression levels (FPKM >1) by mRNA-Seq, 2,358 genes were differentially expressed between tumor and normal tissues (Supplementary Table 2) using two criteria: a greater than 2 fold expression level change and p-value (FDR) ≤ 0.05 from ANOVA test. These 2,358 genes included 1,223 upregulated genes (T/N > 2) and 1,135 downregulated genes (N/T > 2) as shown in the volcano plot (Figure 1A). Unsupervised hierarchical clustering analysis (HCA) of these 2,358 genes showed that 77 normal samples and one low stage tumor sample clustered into a distinct group while 78 tumor samples and 2 normal samples associated with high stage tumors clustered into another group (Figure 1B). Similar results were revealed by a principal component analysis (PCA) (Figure 1C). These data demonstrate that colorectal cancers can generally, but not in every case, be distinguished from their adjacent normal samples using large-scale gene expression analyses.

### Upregulated genes and their corresponding biological processes

**Figure 2:**
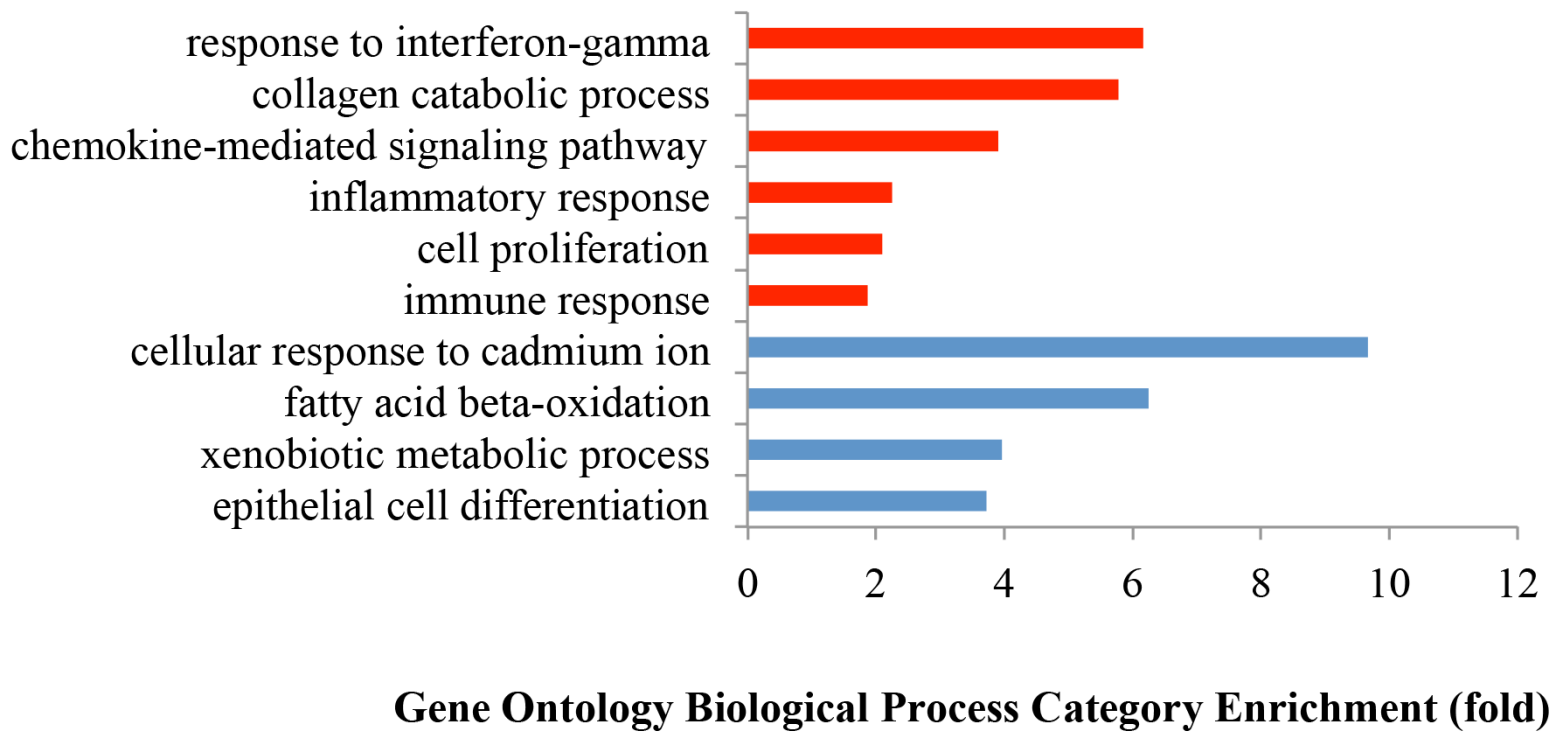
Gene Ontology enrichment analysis of biological processes of the differentially expressed genes between tumors and normal tissues The biological processes were selected using a False Discovery Rate (FDR) < 0.05 in DAVID. Red bars represent the significantly upregulated biological processes with FDR < 0.05; while the blue bars represent the significantly downregulated biological processes with FDR < 0.05.

**Figure 3:**
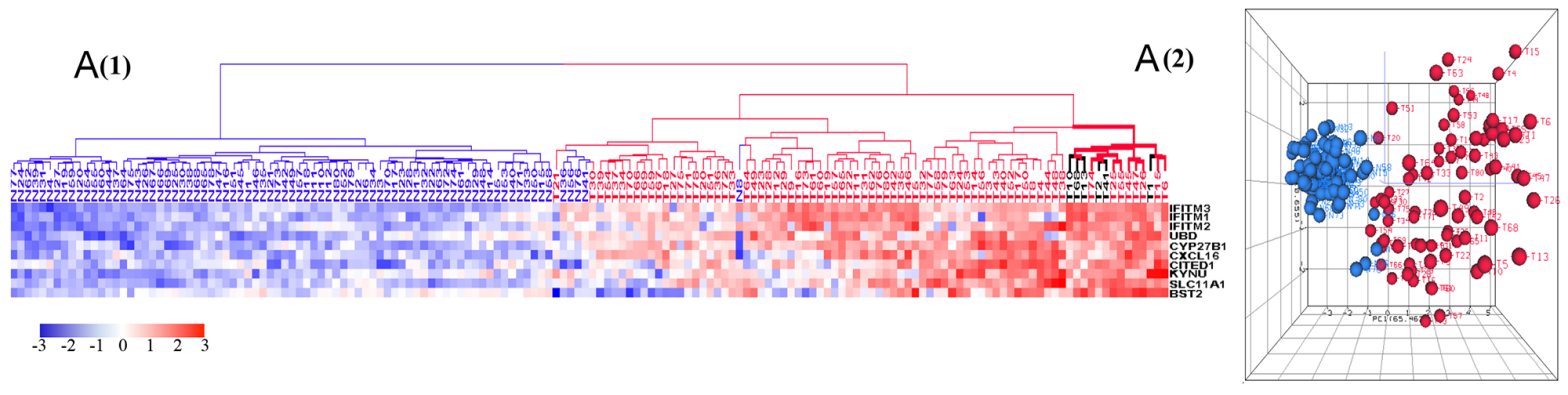

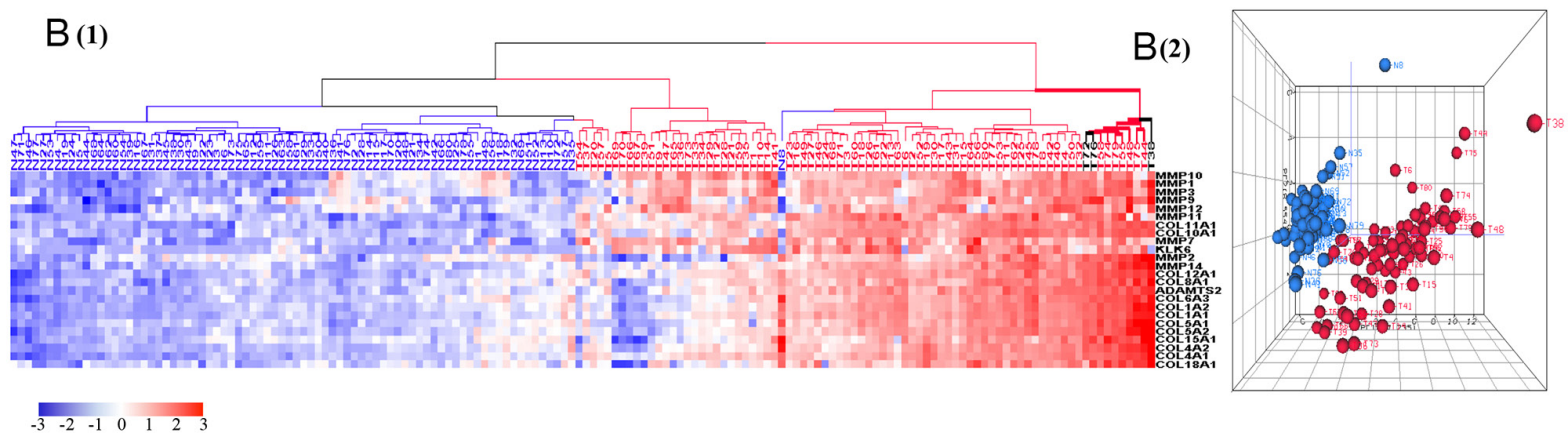

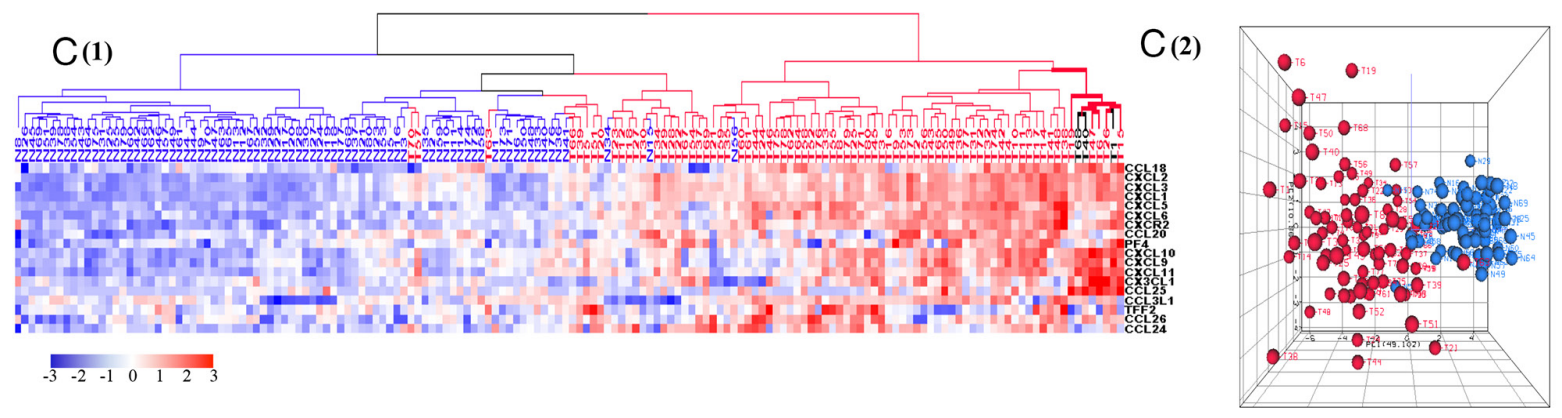

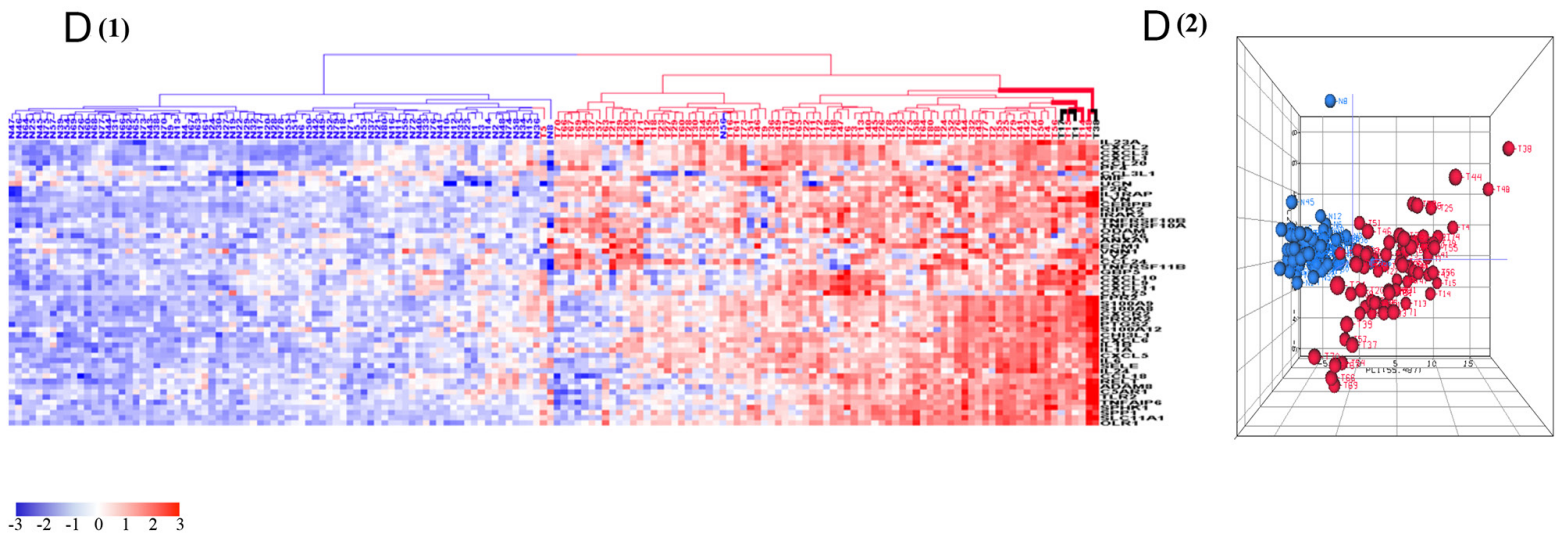

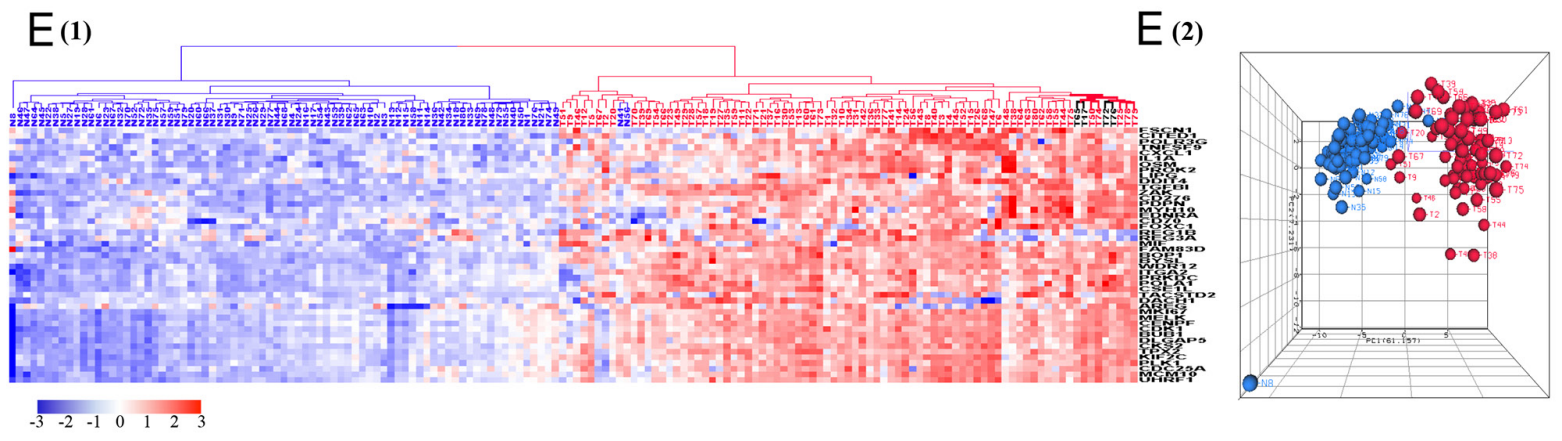

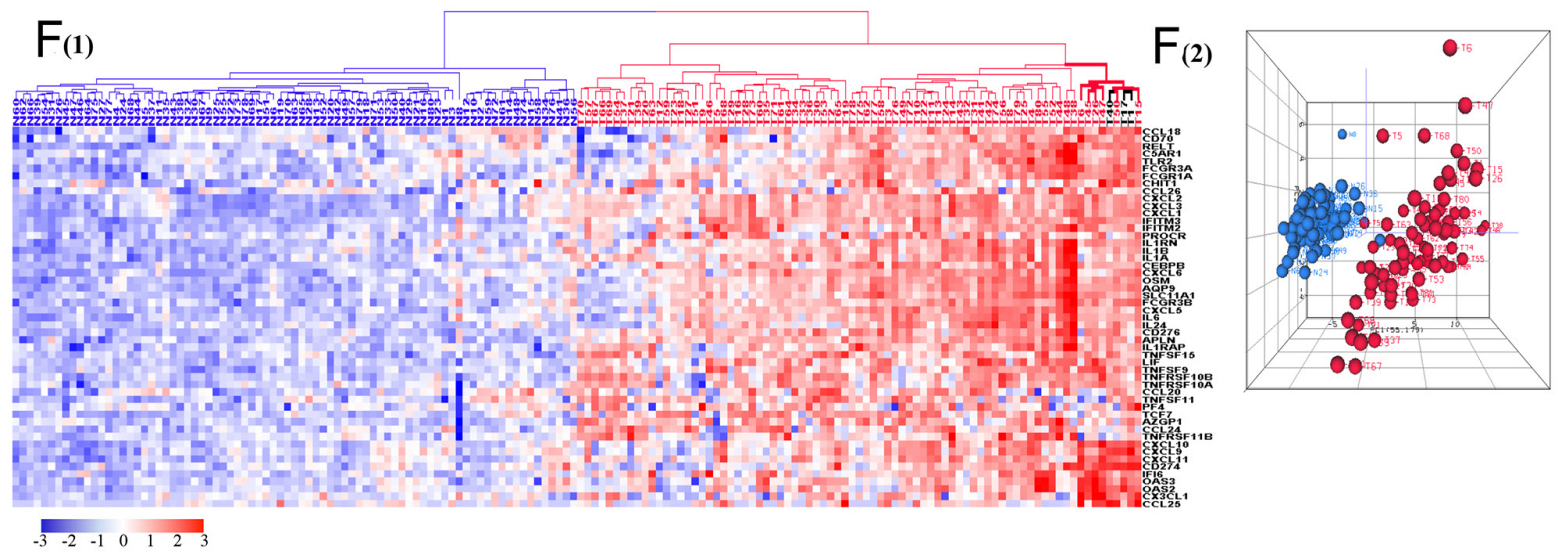
HCA and PCA of genes pertaining to the six most significantly upregulated biological processes in tumors vs. normal tissues These biological processes include response to interferon-gamma **(A)**, collagen catabolic process **(B)**, chemokines mediated signaling pathways **(C)**, inflammatory response **(D)**, cell proliferation **(E)**, immune response **(F)**. Majority of tumors were clustered together within the upregulated side (red) of HCAs. Tumor is marked with red dendrogram and normal sample is marked with blue dendrogram. The bold dendrograms in HCAs mark subgroups of tumors with the greatest upregulated genes. Both high stage tumors (labeled in red) and low stage tumors (labeled in black) are presented in bold dendrograms. Tumors were more heterogeneous than normal controls in PCA plots.

Gene Ontology (GO) analysis of the 1,223 upregulated genes revealed a focus on six key biological processes as defined by DAVID bioinformatics: collagen catabolic process, chemokine-mediated signaling pathway, inflammatory response, cell proliferation, response to IFNγ, and immune response (Figure 2). Since these biological processes principally pertain to tumor environmental alterations (inflammation, proliferation, and invasion), we investigated the differences among tumors of different histologic stages and grades as well as between tumors and normal samples. The clustering results (HCA) revealed that tumors clustered at the high expression zones (red) while normal samples clustered at the low expression zones (blue) (Figure 3). PCA plots mirrored the HCA plots showing moderate heterogeneity among tumors.

The most prominently upregulated genes consisted of families of peptidases, proteinases, and chemokines, including kallikrein-related peptidases (KLKs), matrix metalloproteinase (MMPs), and chemokines. Strikingly, the KLK peptidase genes showed the greatest upregulation: *KLK6* (231 fold increase); *KLK7* (223 fold increase); *KLK10* (43 fold increase); *KLK11* (13 fold increase)*;* and *KLK12* (10 fold increase). In fact, *KLK6* and *KLK7* showed almost no expression in normal samples but have previously been found to be early stage biomarkers for ovarian cancer [11]. Matrix metalloproteinases (MMPs) sharply upregulated in tumors included *MMP7* (126 fold increase), *MMP3* (62 fold increase), *MMP1* (31 fold increase), *MMP10* (13 fold increase), *MMP11* (12 fold increase)*,* and *MMP12* (10 fold increase). KLKs and MMPs are known to be involved in extracellular matrix (ECM) remodeling via degradation of extracellular proteins and collagen matrix. Such space remodeling is necessary for tumor expansion, metastasis and epithelial to mesenchymal transition [12–15]. MMPs also recruit neutrophils by generating bioactive fragments (such as *N*-acetyl Pro-Gly-Pro) from the ECM [16]. Tumor upregulated chemokines included the followings: *CXCL5* (138 fold increase); *CCL25* (70 fold increase); *CXCL11* (26 fold increase); *CXCL6* (20 fold increase); *CXCL1* and *CXCL10* (11 fold increase); *CXCL3* (8 fold increase); and *CXCL9, CXCL2*, and *CCL3L1* (6 fold increase). Other chemokines with lesser upregulation are included in Supplementary Table 2. Upregulated cytokine genes in tumors included *IL11* (26 fold increase), *IL24* (24 fold increase), *IL6* (17 fold increase), *IL1A* (14 fold increase), *IL1B* (10 fold increase), *IL23A* (8 fold increase), and *IL33* (3 fold increase). Intriguingly, although these cytokines are upregulated, the respective receptor genes for *IL1*, *IL6*, *IL11*, and *IL23* are downregulated (N/T > 1.5 fold). Only the receptor gene for *IL24* is upregulated (T/N > 2 fold) within our dataset.

The induction of genes pertaining to multiple chemokines and cytokines suggests a complex tumor microenvironment composed of different cell types that cohabit and communicate with each other via soluble mediators to promote tumor survival, proliferation and metastasis [17, 18]. The high level expression of genes related to macrophages (Supplementary Figure 1A) and neutrophils (Supplementary Figure 1B) further supports this hypothesis. Despite the elevated expression level of chemokine and cytokine genes, there was diminished expression of T and B cell related genes in tumor tissues compared to their normal control tissues (Supplementary Figure 1C). Indeed, the absence of T and B cells in colorectal cancers has been correlated with unfavorable overall survival [19]. The downregulation of T and B cell related genes could be explained by the decreased expression of some chemokines related to the recruitment of T and B cells [20–23], such as *CCL23* (7 fold decrease), *CXCL12* (4 fold decrease), *CCL8* (3 fold decrease), and *CCL13* (2 fold decrease). Additional upregulated genes in tumors that pertain to cell proliferation include those of the regenerating family (REG) genes, such as *REG3A* (23 fold increase), *REG4* (9 fold increase), and *REG1A* (4 fold increase), which are known to be involved in cell proliferation and have a key role in carcinogenesis by activating the AKT and ERK1/2 pathways [24]. As well, upregulated mRNA expression of *REG1A* has been shown as an unfavorable prognostic marker in colorectal cancer and has been associated with peritoneal carcinomatosis [25]. Intriguingly, the mRNA and protein products of these *REG* genes and *CCL25* gene were highly expressed in autoimmune/inflammatory diseases of the intestinal tract including ulcerative colitis (UC), a condition with a high probability of development of colon cancer [26–28].

Well known oncogenes showed the expected upregulation across our samples including *MYC* (4 fold increase), karyopherin alpha 2 (*KPNA2*) (3 fold increase), pituitary tumor transforming gene (*PTTG1*) (2 fold increase), high mobility group AT-hook 1 (*HMGA1*) (3 fold increase), and nucleolar and coiled-body phosphoprotein 1 (*NOLC1*) (3 fold increase). The upregulation of *PHF19* (3 fold increase) which codes for PHD (Cys4-His-Cys motif) finger protein 19, a histone binding protein with embryonic stem cell self-renewal function [29], suggests it may have oncogenic function in colorectal cancer.

### Downregulated genes and their corresponding biological processes

**Figure 4:**
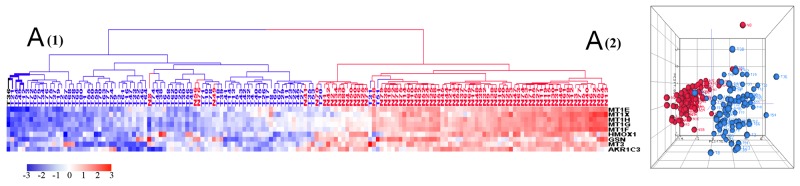

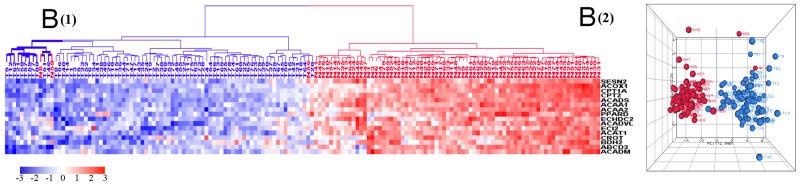

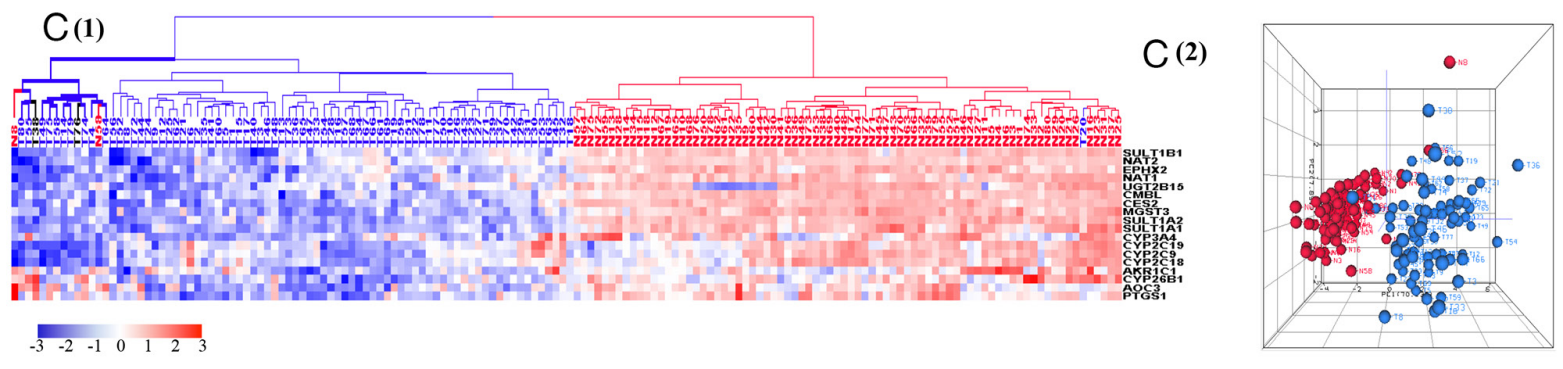

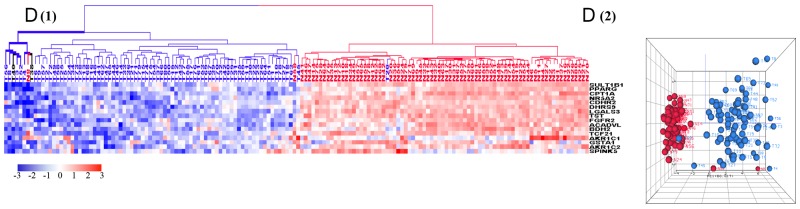
HCA and PCA of genes pertaining to the four significantly downregulated biological processes in tumors vs. normal tissues These biological processes include cellular response to cadmium ion **(A)**, fatty acid beta-oxidation **(B)**, xenobiotic metabolic process **(C)**, and epithelial cell differentiation **(D)**. Majority of tumors were clustered together within the downregulated side (blue) of HCAs. Tumor is marked with blue dendrogram and normal sample is marked with red dendrogram. The bold dendrograms in HCA mark subgroups of tumors with the greatest downregulated genes. Both high stage tumors (labeled in blue) and low stage tumors (labeled in black) are presented in bold dendrograms. Normal controls were more heterogeneous than tumors in PCA plots.

Gene Ontology analysis of 1,135 downregulated genes revealed dramatic differences between tumors and healthy colonic tissue pertaining to four biological processes: fatty acid beta oxidation, colonic detoxification of inorganic heavy metals (such as cadmium), colonic detoxification of organic toxins (such as antibiotics), and colonic epithelium differentiation. Tumors clustered at the low expression zones (blue) while normal samples clustered at the high expression zones (red) of HCAs (Figure 4). The PCA plots, which mirrored the HCA plots, show greater heterogeneity of normal samples compared with tumors in downregulated genes (Figure 4). Five isoforms of carbonic anhydrase (CA), involved in the conversion of CO_2_ and water into bicarbonate and protons, are among the top downregulated genes (Supplementary Table 2): *CA1* (41 fold decrease), *CA7* (25 fold decrease), *CA2* (15 fold decrease), *CA4* (18 fold decrease) and *CA12* (4 fold decrease). These data suggest that the acidic environment generated by tumor cells plays an important role in cell proliferation and metastasis [30] but the signaling pathways and genes that lead to such are not known and require investigation. In contrast to the above carbonic anhydrase genes which were sharply downregulated in tumors, carbonic anhydrase 9 (*CA9*), was overexpressed with a 47 fold expression increase in tumor samples. Upregulation of this *CA* gene is associated with poor prognosis [31].

Downregulation of genes pertaining to membrane transporters included bestrophin 4 (*BEST4*, chloride channel) (42 fold decrease) and solute carrier family genes such as *SLC30A10* (28 fold decrease), *SLC6A19* (23 fold decrease), *SLC26A2* (20 fold decrease), and *SLC26A3* (20 fold decrease). Downregulation of previously reported membrane transporters correlated with the loss of normal intestinal epithelial function in tumor cells [32]. Moderately downregulated genes included Acyl-CoA dehydrogenase (*ACADS*) (5 fold decrease) and Acyl-CoA Dehydrogenase Very Long Chain (*ACADVL*) (2 fold decrease) which are involved in fatty acid beta oxidation.

### Switch of ATP production from mitochondria to cytosol in colorectal cancer

**Figure 5:**
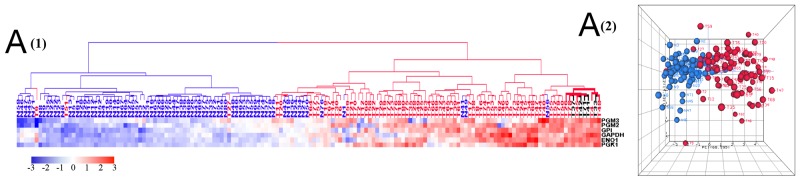

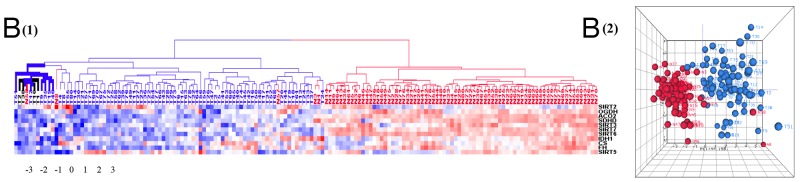

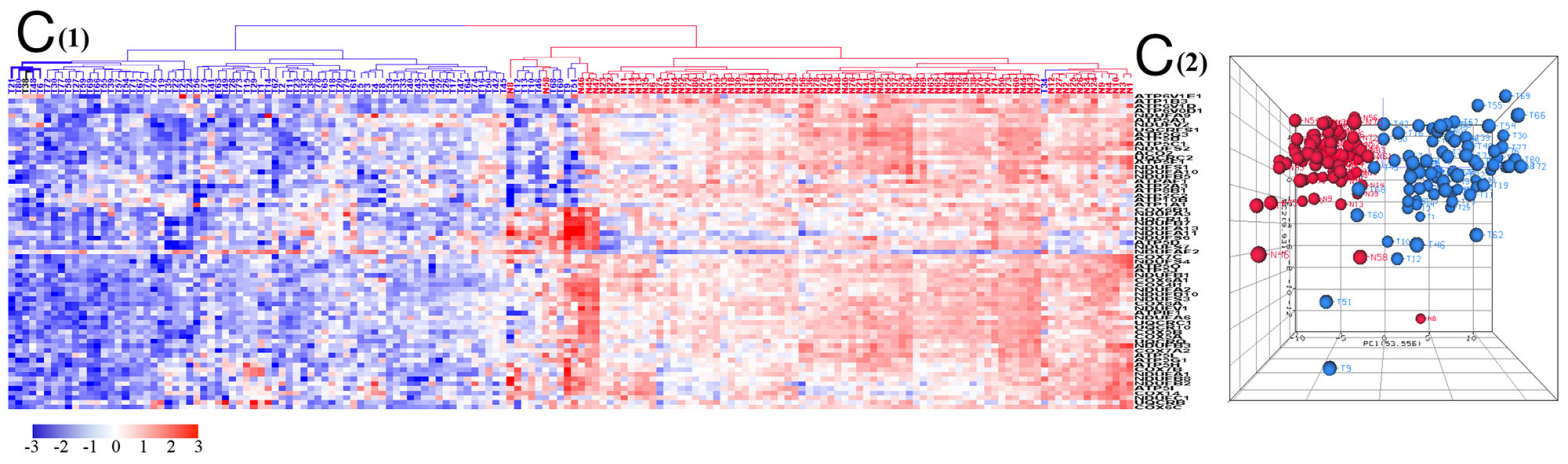
HCA and PCA of genes pertaining to glycolysis **(A)**, TCA cycle and sirtuin pathways **(B)**, and five mitochondrial respiratory chain complexes **(C)**. (A) Majority of tumors were clustered together within the upregulated side (red) of glycolytic HCA. Tumor is marked with red dendrogram and normal sample is marked with blue dendrogram. The bold dendrograms in HCA mark subgroups of tumors with the greatest upregulated corresponding genes. Both high stage tumors (labeled in red) and low stage tumors (labeled in black) are presented in bold dendrograms. Tumors were more heterogeneous than normal controls in PCA plot. (B) and (C) Majority of tumors were clustered together within the downregulated side (blue) of TCA/ SIRT pathway and mitochondrial respiratory chain complexes. Tumor is marked with blue dendrogram and normal sample is marked with red dendrogram. The bold dendrograms in HCAs mark subgroups of tumors with the greatest downregulated corresponding genes. Both high stage tumors (labeled in red) and low stage tumors (labeled in black) are presented in bold dendrograms. Tumors were more heterogeneous than normal controls in PCA plots.

The down-regulation of mitochondrial fatty acid beta-oxidation for ATP production prompted us to examine other energy generating pathways in tumor tissues including glycolysis, the tricarboxylic acid (TCA) cycle, glycolysis suppressive Sirtuin (*SIRT)* pathways, and the mitochondrial respiratory chain. Genes related to glycolysis, TCA/SIRT pathways, and the mitochondrial respiratory chain were identified from the 10,255 gene data set with an average FPKM >1 and FDR ≤ 0.05 in ANOVA. For glycolysis related genes, tumors clustered at the high expression zone (red) while normal samples clustered at the low expression zone (blue) (Figure 5A). The genes involved in the SIRT pathway, TCA cycle, and mitochondrial respiratory chain related functions were sharply downregulated with tumors clustered at the low expression zone in dark blue and normal samples at the high expression zone in red (Figure 5B, 5C). The PCA plots showed similar results with tumors separated from normal samples (Figure 5). The downregulated fatty acid oxidation, TCA/SIRT pathway, and mitochondrial respiratory chain pathway, as well as upregulated glycolysis support the classical Warburg effect [5–7] in colorectal cancer as in other tumor tissues [33–36].

### Determination of gene co-expression network and hub genes in colorectal cancer

**Figure 6:**
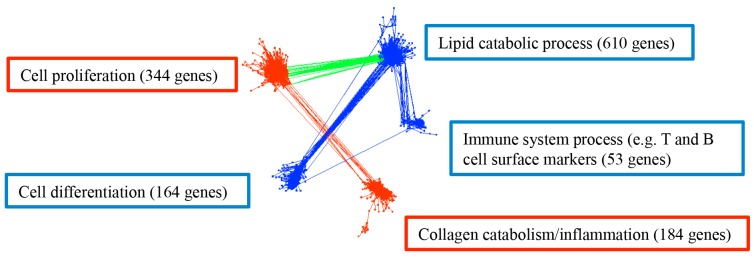
Gene co-expression network in colorectal cancer with 1,355 genes and 54,018 connections Upregulated biological processes for the corresponding genes are labeled in red boxes and downregulated biological processes are in blue boxes. Red edges are the connections between upregulated processes, blue edges are the connections between downregulated processes, and green edges are the connections between up- and down-regulated processes. Gene number in each module is presented in each corresponding box. The representative biological processes were determined in DAVID with FDR < 0.05.

To explore possible interactions of deregulated biological processes, we generated a comprehensive gene co-expression network from the identified 2,358 deregulated genes. This co-expression network is composed of 1,355 genes with a total of 54,018 strong connections as defined by the threshold value of > 0.8 for the absolute value of the Pearson’s correlation coefficient (cc) between the expression levels of two genes (Figure 6). Two upregulated modules included 344 cell proliferation related genes as well as 184 collagen catabolism and inflammation related genes; while 3 downregulated modules included 610 lipid catabolic process related genes, and 164 cell differentiation related genes, as well as 53 genes pertaining to T and B cell related immune system process including *LY9, CR2, CXCR5, CD19, CD79A,* and *CD79B*. The following correlations among modules were observed: first, lipid catabolic process positively correlated (cc > 0.8) with cell differentiation and T cell / B cell related immune system process; second, lipid catabolic process negatively correlated (cc < -0.8) with cell proliferation; third, cell proliferation strongly positively correlated (cc > 0.8) with collagen catabolism and inflammation (Figure 6). Although the increased de novo synthesis of fatty acids has been reported in various cancer cells [37, 38], the association of decreased lipid catabolism, such as fatty acid beta-oxidation, with cell proliferation, dedifferentiation and diminished lymphocyte markers in tumor is a novel finding. Similarly, cell proliferation, collagen catabolism and inflammation have been recognized as hallmarks of cancers [12–16, 39], but the correlated dysregulation with respect to metabolic and differentiation parameters has not previously been reported.

**Figure 7:**
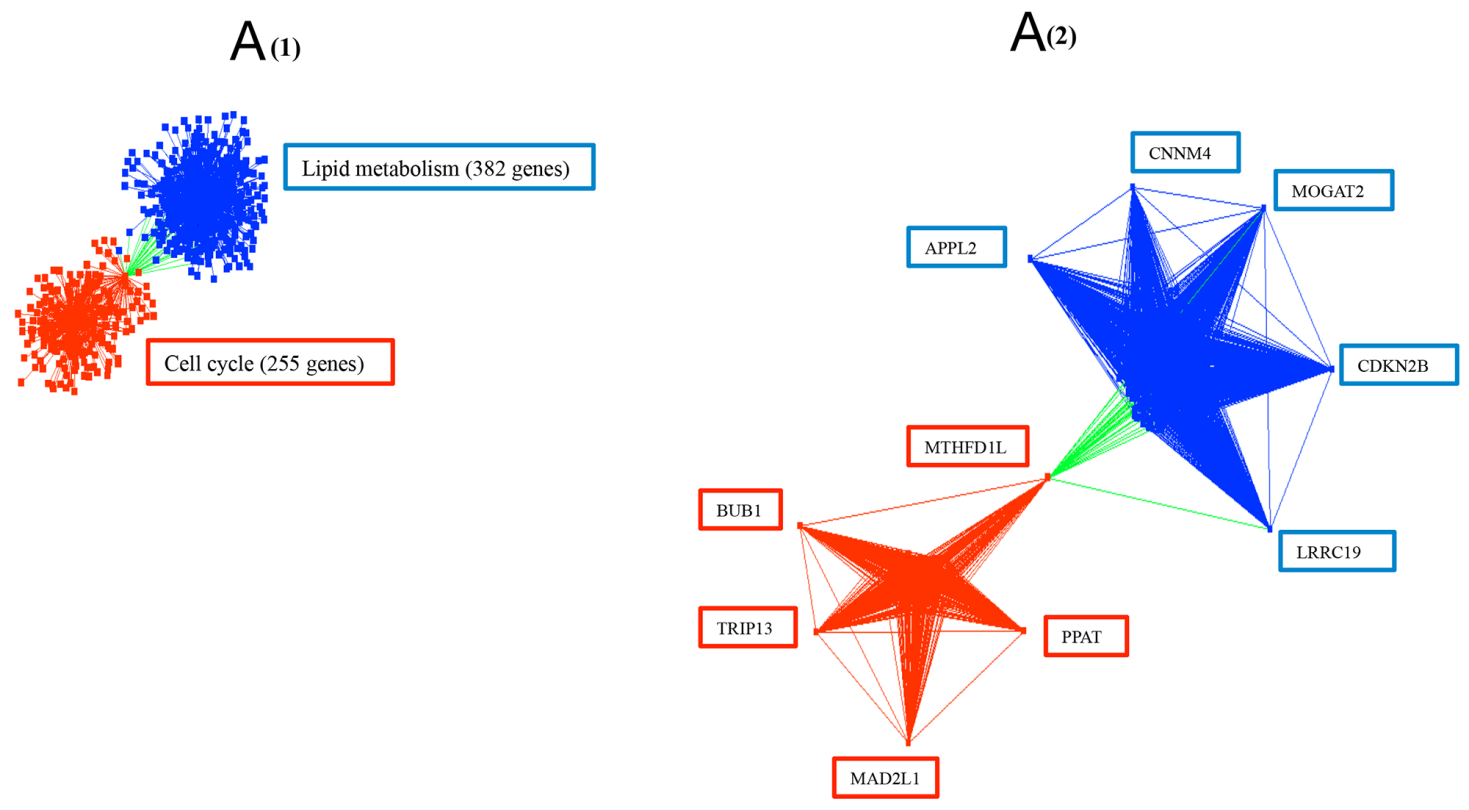

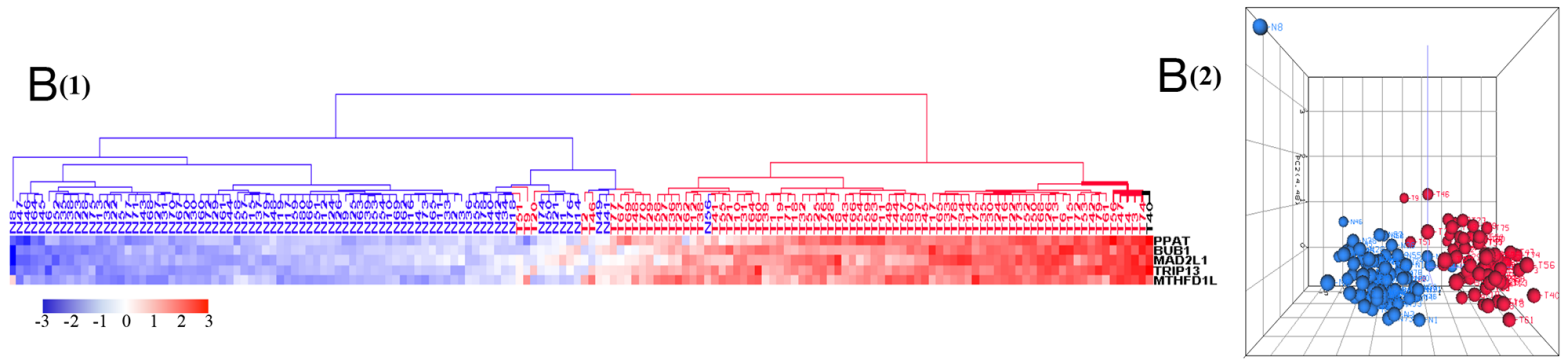

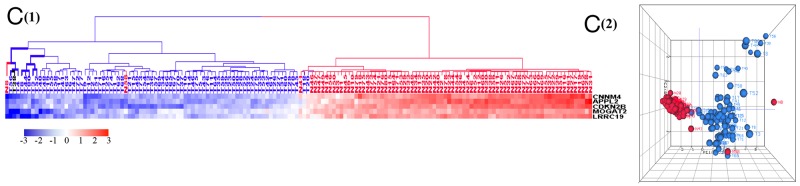
**(A)** The connections of ten potential hub genes and their correlated 607 genes with 2,080 interactions. The upregulated hub genes are in red squares and the downregulated hub genes are in blue squares. Red edges are the connections between upregulated genes, blue edges are the connections between downregulated genes, and green edges are the connections between up and down regulated genes. The represented biological processes for the corresponding genes, determined in DAVID with FDR < 0.05, are labelled in boxes. **(B)** and **(C)** HCA and PCA of the upregulated hub genes and downregulated hub genes. The five upregulated hub genes showed increased expression in 76 out of 79 tumors, and the five downregulated hub genes showed decreased expression in 77 out of 79 tumors when compared with normal samples. (B) Tumor is marked with red dendrogram and normal sample is marked with blue dendrogram. The bold dendrograms in HCAs mark subgroups of tumors with the greatest upregulated hub genes. Both high stage tumors (labeled in red) and low stage tumors (labeled in black) are presented in bold dendrograms. (C) Tumor is marked with blue dendrogram and normal sample is marked with red dendrogram. The bold dendrograms in HCAs mark subgroups of tumors with the greatest downregulated hub genes. Both high stage tumors (labeled in blue) and low stage tumors (labeled in black) are presented in bold dendrograms.

To probe for candidate hub genes we identified 5 up and downregulated genes with the greatest number of co-expressed genes (from 106 to 383 genes) (Table 1). Five upregulated candidate hub genes included methylenetetrahydrofolate dehydrogenase (NADP+ dependent) 1-like (*MTHFD1L*), mitotic checkpoint serine/threonine kinase (*BUB1*), thyroid hormone receptor interactor 13 (*TRIP13*), mitotic spindle assembly checkpoint protein (*MAD2L1*), and phosphoribosyl pyrophosphate amidotransferase (*PPAT*). Five downregulated putative hub genes included amyloid beta precursor like protein 2 (*APPL2*), cyclin M4 (*CNNM4*), monoacylglycerol O-acyltransferase 2 (*MOGAT2*), cyclin-dependent kinase inhibitor 2B (*CDKN2B*), and leucine rich repeat containing 19 (*LRRC19*). In the co-expression network, the upregulated hub genes correlated with 225 genes related to cell cycle, and the downregulated hub genes correlated with 382 genes related to lipid metabolism (Figure 7). Interestingly, *MTHFD1,* an enzyme involved in the synthesis of tetrahydrofolate (THF) within the mitochondrion [40], correlated positively with upregulated genes and negatively with downregulated genes, thus acting as a potential link between cell cycle and lipid metabolism.

**Table 1:** Five upregulated hub genes and five downregulated hub genes

Upregulated hub genes	Proteins coded	Gene function	Expression fold change (T/N)	Number of correlated genes (|CC| ≥ 0.8)
MTHFD1L	methylenetetrahydrofolate dehydrogenase (NADP+ dependent) 1-Like	de novo synthesis of purines and thymidylate	5.51	176
BUB1	mitotic checkpoint Serine/Threonine kinase	activate spindle checkpoint	3.15	150
TRIP13	thyroid hormone receptor interactor 13	inactivate spindle assembly checkpoint	4.09	145
MAD2L1	mitotic arrest deficient 2 like 1	mitotic spindle assembly checkpoint protein	3.08	109
PPAT	phosphoribosyl pyrophosphate amidotransferase	de novo synthesis of purines	3.06	106

Downregulated hub genes	Proteins coded	Gene function	Expression fold change (T/N)	Number of correlated genes (|CC| ≥ 0.8)
APPL2	amyloid beta precursor like protein 2	cell proliferation regulation	0.29	383
CNNM4	cyclin M4	metal transporter	0.33	365
MOGAT2	monoacylglycerol O-Acyltransferase 2	fat digestion and absorption	0.16	361
CDKN2B	cyclin-dependent kinase inhibitor 2B	inhibit cell cycle G1 progression	0.14	357
LRRC19	leucine rich repeat containing 19	Membrane protein	0.17	357

## DISCUSSION

In this study, we used high-throughput mRNA-Seq technology to examine the whole transcriptome in a total of 79 colon cancers and matched normal tissue controls. Our study provides a comprehensive gene expression analysis in colon cancer tissues. The upregulated response to IFNγ, inflammation response, immune and chemokine mediated signaling pathways (Figure 2, 3) demonstrate that inflammation is a major feature of the tumor microenvironment in colorectal cancer. Interferon induced transmembrane proteins (*IFITMs),* can be transcriptionally induced by type I and type II interferons [41]. We observed that tumor samples had upregulated *IFITMs* including *IFITM1, IFITM2,* and *IFITM3* (Figure 3), consistent with previous observations in colorectal cancer [42–44]. Although it has been used to treat tumors due to its immunostimulatory function, paradoxically IFNγ, has recently been found to promote tumor progression by upregulating checkpoint inhibitors [45, 46]. This tumor-promoting function for IFNγ is supported by the upregulated IFNγ (Supplementary Figure 2) observed in our study. Such activity, however, may lead to favorable responses to checkpoint inhibition in this setting [47, 48].

Five of 9 metallothionein (*MT*) genes were downregulated in tumors (Figure 4A). The MT proteins are capable of binding heavy metal (cadmium, mercury, lead, and arsenic) and their diminished expression is associated with poorer survival in colorectal cancer [49]. We also found that 5 of 22 cytochrome P450 (*CYP*) genes, whose associated enzymes oxidize small foreign organicmolecules such as toxins or drugs [50], were downregulated in tumors (Figure 4C). These findings suggest that colorectal cancer cells lose key physiological functions of detoxification which could be the result of epithelial cell dedifferentiation (Figure 2, 4) [51]. These findings are in keeping with observations that colonic dedifferentiation may initiate intestinal tumorigenesis [52].

The identification of highly connected potential hub genes from the gene co-expression network should facilitate the identification of candidate biomarkers and therapeutic targets for colorectal cancer. Among 10 putative hub genes (Table 1, Figure 7), all 5 upregulated hub genes have been identified as oncogenes [40, 53–59], and all 5 downregulated hub genes have been identified as tumor suppressor genes [60–64]. *MTHFD1L* is the only hub gene which has both positive and negative correlations with genes pertaining to cell cycle and lipid metabolism respectively (Figure 7A). Specifically, the MTHFD1L enzyme is involved in the synthesis of tetrahydrofolate (THF) within the mitochondrion [40], in contrast to cytosolic methylene tetrahydrofolate reductase (MTHFR), a drug target of methotrexate (MTX) [65]. Inhibition of mitochondrial *MTHFD1L* expression specifically in tumors may thus provide a new therapeutic target. Overexpression of *MAD2L1* and *BUB1*, candidate hub genes in our study, has previously been associated with high grade breast tumors and poor survival of breast cancer patients [66, 67]. *TRIP13,* critical for the inactivation of the spindle assembly checkpoint, is associated with the progression of certain cancers, and was found to be highly expressed in multiple colorectal cancer tissues as assessed by RT-PCR [58]. *PPAT* gene was identified as a prognostic biomarker in aggressive lung adenocarcinoma by RNA-Seq analysis [68]. These overexpressed potential hub genes may have relevance as diagnostic or prognostic biomarkers for colorectal cancer. Of note, the diminished expression of putative hub genes such as *CDKN2B, CNNM4, LRRC19,* and *MOGAT2* might be the result of inactivation of genes through DNA methylation as their methylation was identified in colorectal, gastric, and breast cancers as well as in leukaemia [62, 69–71]. The DNA demethylation agent arsenic trioxide was able to restore the expression of methylation-silenced *CDKN2B* in the human T lymphoblast cell line Molt4 and human myelodysplastic syndrome-(MDS-REBT) cell line Mutz-1 [72], indicating the potential of DNA demethylation agents to contribute to cancer treatment in some instances.

Not to be overlooked, in our analyses we observed that a few normal controls clustered with high stage and low stage tumors while multiple low stage tumors clustered with high stage tumors in certain biological pathways (Figure 3, 4, 5, 7, Table 2A). These discrepancies between histological determination and molecular profiles suggest the necessity of molecular subtyping of colorectal cancers pertaining to numerous attributes correlated with tumor invasiveness and metastasis [73, 74]. Moreover, the so-called normal tissues adjacent to either high or low stage tumors in the heatmaps (Table 2B) showed molecular profiles of malignancy, indicating the presence of either histologically undetected tumor budding or altered metabolism imposed by proximal tumors. As the tumor margin determines “surgical clearance” which, in turn, impacts the potential for both local recurrence and overall survival [75-77], a molecular evaluation of tumor adjacent tissues rather than sole reliance on a histological determination may better determine true negative margins.

In summary, in applying mRNA-Seq to analyze transcriptome profiles in 79 primary colorectal cancers which were stringently collected, stored and processed, we showed that colorectal cancer cells/tissues exhibit malignant features such as cell proliferation, tissue remodeling and cytokine related inflammation and lose the physiological functions of normal colorectal tissue such as metabolism of toxins. Our comprehensive co-expression network provides a global view of interacting biological processes in colorectal cancer which should facilitate discovery of tumor specific targets for the treatment of this deadly disease.

**Table 2A:** Discrepancy between histological diagnosis and molecular profiles

Dysregulated biological processes	A few tumors clustered with most normal samples		A few normal samples clustered with most tumors	
	High stage tumors	Low stage tumors	Normal samples adjacent to high stage tumors	Normal samples adjacent to low stage tumors
upregulated response to interferon-gamma (10 genes)	0	0	N8	N36, N41, N66, N76
upregulated collagen catabolic process (24 genes)	0	0	N8	0
upregulated chemokines mediated signaling pathways (18 genes)	T57, T63	T70	N15, N34, N56	0
upregulated inflammatory response (56 genes)	0	0	N8	0
upregulated cell proliferation (50 genes)	0	0	N56	N41
upregulated immune response (51 genes)	0	0	0	0
upregulated glycolysis (6 genes)	T9, T51, T27	0	N56, N49, N50, N21, N34, N2	N1, N18, N36, N41, N42, N76
upregulated hub genes (5 genes)	T9, T46, T51	T2, T20	N56	0
downregulated cellular response to cadmium (9 genes)	T51	T20, T31	N8, N49, N56	N41, N76
downregulated acid beta-oxidation (16 genes)	0	0	N8, N56, N58	0
downregulated xenobiotic metabolic process (18 genes)	0	T20	N8, N58	0
downregulated epithelial cell differentiation (16 genes)	0	T20	N8, N58	0
downregulated SIRT and TCA cycle (11 genes)	0	0	N3, N8, N57, N58	N1
downregulated respiratory chain (67 genes)	T34	0	N8, N58	0
downregulated hub genes (5 genes)	0	0	N8, N58	N41

**Table 2B:** Low stage tumors and normal samples show similar gene expression profiles with high stage tumors.

Dysregulated biological processes	Tumors and normal samples clustered within high expression zones		
	High stage tumors	Low stage tumors	Normal samples adjacent to high stage tumors
upregulated response to interferon-gamma	T6, T14, T16, T25, T26, T46, T47, T55	T1, T2, T10, T13, T41, T68	0
upregulated collagen catabolic process	T44, T48, T55, T56, T74, T75, T79	T38, T72, T76	0
upregulated chemokines mediated signaling pathways	T6, T15, T26, T47	T1, T40, T68	0
upregulated inflammatory response	T14, T15, T48	T1, T17, T38	0
upregulated cell proliferation	T25, T74, T50, T78, T79	T17,T65, T72, T76	0
upregulated immune response	T6, T15,, T26, T45, T47, T50	T1, T17, T40	0
upregulated glycolysis	T4, T26, T44, T45, T48, T75	T33, T37, T41, T42, T71	0
upregulated hub genes	T3, T47, T56, T74	T40	0

	Tumors and normal samples clustered within low expression zones		
Dysregulated biological processes	High stage tumors	Low stage tumors	Normal samples adjacent to high stage tumors
downregulated acid beta-oxidation	T9, T12, T21, T46, T51, T62, T57, T58, T59, T77, T80	T36	N8, N58
downregulated xenobiotic metabolic process	T4, T5, T35, T8, T14, T49, T52, T56, T58, T74, T75, T79, T80	T38, T76	N8, N58
downregulated epithelial cell differentiation	T3, T4, T8, T52, T56	T38, T40	N8
downregulated SIRT and TCA cycle	T9, T12, T19, T35, T48, T51	T1, T2, T10, T38	N8, N58
downregulated respiratory chain	T6, T21, T48, T80	T38	0
downregulated hub genes	T3, T4, T8, T52, T56, T80	T38	N8

## MATERIALS AND METHODS

### Cohort

Seventy-nine paired-tissues (79 tumor and 79 normal controls, Supplementary Table 1) of pretreatment colorectal cancers were collected from 38 male and 41 female patients by Indivumed GmbH (Germany) for mRNA sequencing. To evaluate tumor content, hematoxylin and eosin stained microscopic slices were examined by pathologists to determine the tumor cell and normal cell areas, respectively. Histologically, tumor content is 50-70% in tumors and 0% in normal tissues. Normal tissues were collected from a site at a minimum of 5 cm from the tumor margin. Ischemia time to freeze was 6-11 minutes. The short cold ischemia increases the likelihood that the data do not relate to postsurgical tissue processing artifacts [8]. According to the medical pathology report, tumors were classified as well, moderately, and poorly differentiated tumors following international guideline UICC TNM-classification [9]. For the convenience of analysis, 26 tumors with stage 1 and 2 were considered as low stage tumors, while 53 tumors with stage 3 and 4 as well as lymph node (LN) and lymphatic vessel (LV) positive were considered as high stage tumors. The ratio of high stage tumors vs. low stage tumors is 2 to 1. There were 17 well (low grade) differentiated, 36 moderate (medium grade) differentiated, and 26 poorly (high grade) differentiated tumors. Clinical and histopathological characteristics of the patients as well as tumor location are summarized in Supplementary Table 1. Among these 80 tumor pairs, 79 pairs were sequenced except the T7/N7 pair.

### mRNA sequencing

RNA quality was assessed using the Agilent 2100 Bioanalyzer, with cellular RNA analyzed using the RNA 6000 Nano Kit (Agilent). Samples with a RNA Integrity Number (RIN) of 7 or higher were processed to generate libraries for mRNA sequencing following the Illumina® TruSeq Stranded mRNA Sample Preparation Guide. In this method, poly (A) RNA was purified from 0.5 μg total RNA, fragmented and reverse-transcribed into cDNAs. Double strand cDNAs were adenylated at the 3’ ends and ligated to indexed sequencing adaptors, followed with briefly amplification for 15 cycles. One femtomole of the sequencing libraries (median size ∼260nt) were denatured and loaded onto a flow cell for cluster generation using the Illumina cBot. Every six samples were loaded onto each lane of a rapid run flow cell. Paired-end sequencing was carried out on HiSeq 2500 sequencer (Illumina, San Diego, CA, USA) for 100 x 2 cycles. For each sample, we obtained ∼50 million 100-bp reads that passed preset filtering parameters.

### Sequencing data analysis

For mRNA sequencing, Tophat V.2.0.11 and Cufflinks V.2.2.1 were used to align the short reads in fastq files to the RefSeq UCSC human hg19 transcript reference genome annotation database and the quantification of relative abundance of each transcript was reported as fragments per kilobase of transcript per million mapped reads (FPKM). ANOVA test was conducted (on Partek genomics suite) to identify mRNAs with differential expression between tumors and matched normal adjacent tissues using the threshold False Discovery Rate (FDR) ≤ 0.05. Genes with at least 2 fold change (increase or decrease) of expression levels as well as FDR ≤ 0.05 in ANOVA test were selected as significantly dysregulated genes between tumor and normal tissues. These genes were used for correlation analysis by Partek. Genes with Pearson’s correlation coefficient (cc) > 0.8 or < -0.8 were used for network construction in Cytoscape (2.8.2). The genes with the greatest number of correlated genes were defined as potential hub genes. The unsupervised hierarchical clustering analysis (HCA) and principal component analysis (PCA) were used to explore the gene expression profiles in ArrayTrack (The National Center for Toxicological Research, U.S. Food and Drug Administration).

### Colorectal cancer gene expression landscape

A total of 25,761 genes were detected and 17,055 genes were differentially expressed (FDR ≤ 0.05 in ANOVA). There were 9,211 genes (35% of total genes) with average FPKM (across 158 samples) < 1 and 16,550 genes (65% of total genes) with average FPKM > 1. Since relative higher FPKM values generally confer more reliable quantitation of the genes in samples [10], our data analysis focused on 10,255 genes (40% of total genes) with average FPKM >1 and differential expression between tumor and normal controls (FDR ≤ 0.05 in ANOVA).

### Gene ontology and KEGG pathway analysis

Gene ontology (GO) analysis was performed on genes with differential expression between tumor and normal samples using the Database for Annotation, Visualization, and Integrated Discovery (DAVID) v6.8 (https://david.ncifcrf.gov/), NIAID/NIH. False Discovery Rate (FDR) < 0.05 was used as the criteria for GO category enrichment.

### Ethics statement

Written informed consent was obtained from all participants involved in the study via the procuring laboratory at Indivumed (Hamburg, Germany).

## SUPPLEMENTARY MATERIALS FIGURES AND TABLES






