# Supplementary material for: Transcriptome analysis of human colorectal cancer biopsies reveals extensive expression correlations among genes related to cell proliferation, lipid metabolism, immune response and collagen catabolism
Source: Oncotarget. 2017 Aug 18;8(43):74703–19. doi: 10.18632/oncotarget.20345 (PMC5650373; doi:10.18632/oncotarget.20345)
Supplement: Supplementary file 1 [file oncotarget-08-74703-s001.pdf]

Transcriptome analysis of human colorectal cancer biopsies reveals extensive expression correlations among genes related to cell proliferation, lipid metabolism, immune response and collagen catabolism

SUPPLEMENTARY MATERIALS

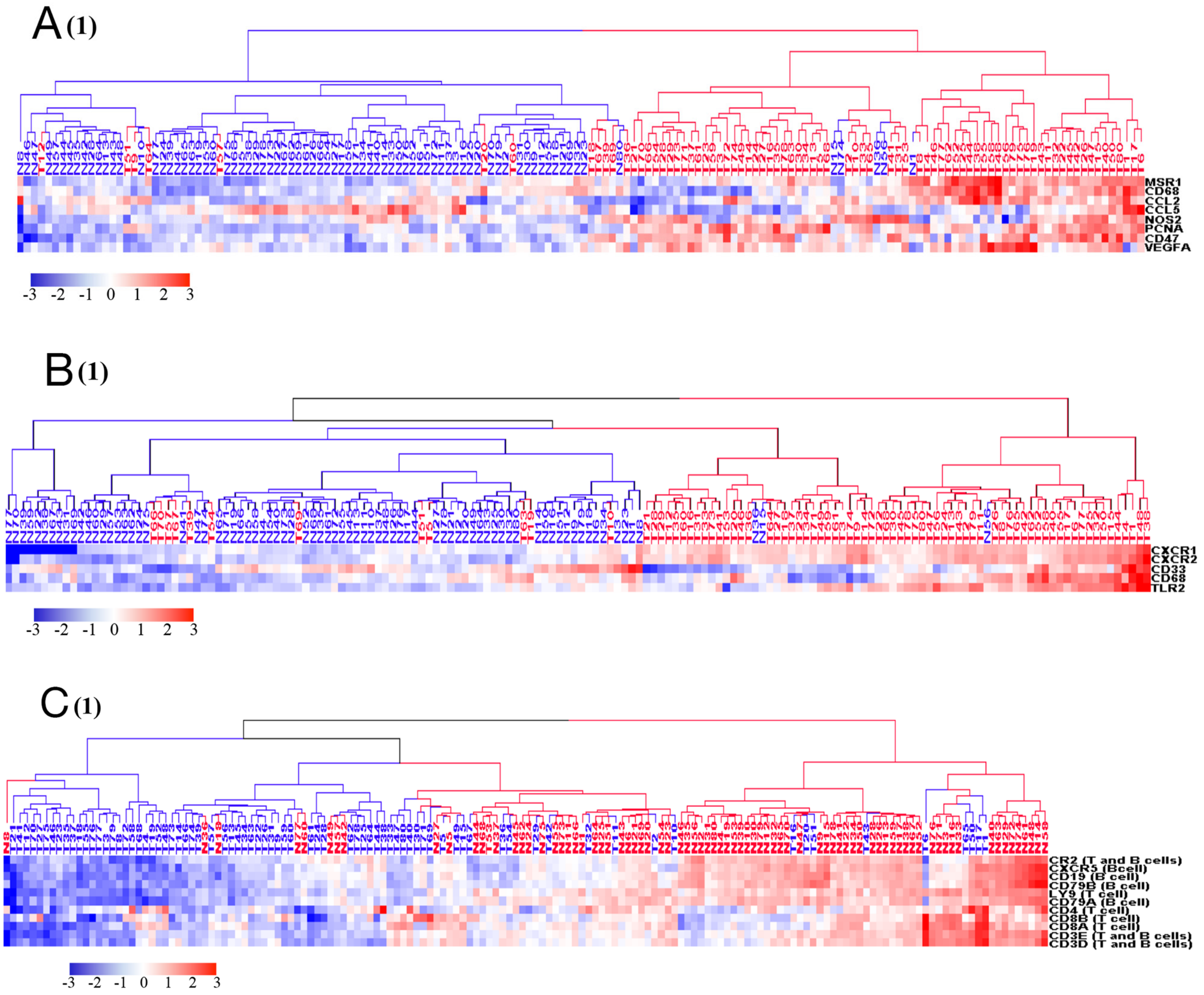

Supplementary Figure 1: Dysregulation of immune cell markers in colorectal cancers. **(1A)** Seventy-two tumors showed upregulation of 8 M2 macrophage related genes compared with normal controls. **(1B)** Sixty-seven tumors had upregulation of 5 neutrophil related genes compared with normal controls. **(1C)** Sixty-one tumors downregulated 11 T cell / B cell related genes compared with normal controls.

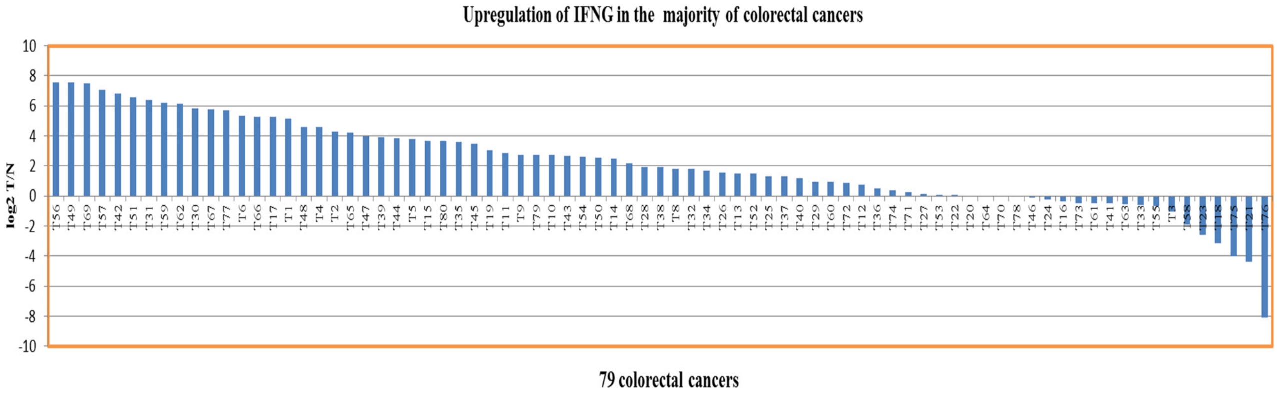

Supplementary Figure 2: Upregulation of *IFNγ* (*IFNG*) in many colorectal cancer samples. Fifty-nine tumors showed upregulation of *IFNγ*, 16 tumors showed downregulation of *IFNγ*. The remaining four tumors did not show *IFNγ* expression change or no *IFNγ* expression was quantified.

Supplementary Table 1: Histological information for 80 colorectal cancers. All tumor samples and associated normal control samples were provided by Indivumed Inc. Tumors are presented from stage 1 to stage 4. For tumor grade, W indicates well differentiated tumor, M indicates moderately differentiated tumor, and P indicates poorly differentiated tumor.

See Supplementary File 1

Supplementary Table 2: 2,358 genes showed significant differential expression between tumors and normal tissues using two criteria: greater than 2 fold expression level change and a $p \leq 0.05$ from paired t-test. These 2,358 genes include 1,223 upregulated genes and 1,135 downregulated genes. FPKM_ave is the mean of FPKM values in all samples. In fold change column, positive number indicates upregulation of gene expression and negative number indicates downregulation of gene expression. The 69 genes in red were previously identified in the meta-analysis of colorectal cancers (76, 77).

See Supplementary File 2
